# Role of Nanomedicine in Transforming Pharmacotherapy for Substance Use Disorder (SUD)

**DOI:** 10.1002/wnan.70008

**Published:** 2025-04-07

**Authors:** Akshata Y. Patne, Subhra Mohapatra, Shyam S. Mohapatra

**Affiliations:** ^1^ Center for Research and Education in Nanobioengineering, Department of Internal Medicine, Morsani College of Medicine University of South Florida Tampa Florida USA; ^2^ Graduate Programs, Taneja College of Pharmacy, MDC30, 12908 USF Health Drive Tampa Florida USA; ^3^ Department of Molecular Medicine, Morsani College of Medicine University of South Florida Tampa Florida USA; ^4^ Research Service James A. Haley Veterans Hospital Tampa Florida USA

**Keywords:** alcohol use disorder (AUD), cannabis use disorder (CUD), immunotherapy, nanomedicine, neurostimulation, opioid use disorder (OUD), personalized medicine, pharmacotherapy, stimulant use disorder, substance use disorder (SUD)

## Abstract

The field of nanomedicine offers revolutionary potential to reshape the discovery and development of therapeutics for diverse human diseases. However, its application has been limited in improving Substance Use Disorders (SUDs), which represent a profound public health crisis, including major types such as opioid, alcohol, stimulant, and cannabis use disorders. Pharmacotherapy, a cornerstone of SUD management, has reduced morbidity, mortality, and the societal impact of addiction, though its efficacy has ranged from none to moderate. Thus, there is a major unmet need to transform SUD pharmacotherapy to curb the epidemic of addiction. This article explores the potential roles of nanomedicine‐inspired precision‐targeted drug delivery, sustained release, and combination therapies to increase therapeutic efficacy and minimize side effects. Additionally, it discusses innovative mechanisms that align with the neurobiological complexities of addiction and synergistic approaches that integrate nanomedicine with behavioral interventions, device‐based therapies, and emerging modalities such as immunotherapy and neurostimulation. Despite these advancements, barriers such as treatment accessibility, adherence challenges, and inequitable resource distribution persist, particularly in underserved populations. By harnessing the transformative capabilities of nanomedicine and integrating it into holistic, equitable, and personalized care frameworks, this review highlights a path forward to revolutionize the SUD pharmacotherapy landscape. The article underscores the need for continued nano‐SUD pharmacotherapy research and the development of strategies to alleviate the substantial burden of addiction on individuals, families, and society.

## Graphical abstract

Nanotechnology‐Driven Innovations in Substance Use Disorder (SUD) Management: Multifunctional Nanotherapeutics Targeting the Brain.

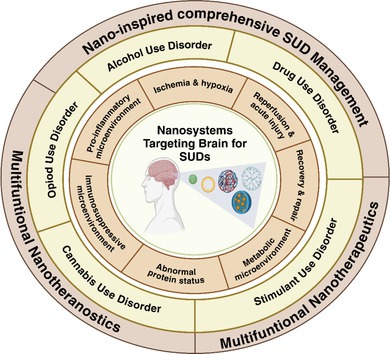

**Abbreviations**
ADHD: attention‐deficit hyperactivity disorder
AUD: alcohol use disorder
BBB: blood–brain barrier
CBT: cognitive behavioral therapy
COMT: catechol‐O‐methyltransferase
CRISPR: clustered regularly interspaced short palindromic repeats
CUD: cannabis use disorder
CYP2D6: Cytochrome P450 2D6
CYP3A4: Cytochrome P450 3A4
DBS: deep brain stimulation
DDS: drug delivery system
DRD2: dopamine receptor D2
DUD: drug use disorder
FAAH: fatty acid amide hydrolase
FDA: Food and Drug Administration
LNP: lipid nanoparticle
MAT: medication‐assisted treatment
MENP: magnetoelectric nanoparticle
MNP: magnetic nanoparticle
NP: nanoparticle
OPRM1: opioid receptor Mu 1
OUD: opioid use disorder
PCR: polymerase chain reaction
qPCR: quantitative polymerase chain reaction
siRNA: small interfering RNA
SUD: substance use disorder
TMS: transcranial magnetic stimulation

## 1 Introduction

Substance use disorder (SUD) represents a critical public health crisis in the United States, significantly affecting individuals, families, and broader society. Defined as a chronic and relapsing brain disease, SUD emerges from a complex interplay of genetic, neurobiological, and environmental factors, manifesting as compulsive drug‐seeking behavior despite harmful consequences (Volkow and Blanco 2023). Its broad spectrum includes opioid use disorder (OUD), alcohol use disorder (AUD), and stimulant use disorders, each presenting unique treatment challenges. Tailored, evidence‐based interventions are essential to achieve effective outcomes. The growing recognition of SUD's multifaceted nature has spurred significant research into novel therapeutic approaches, particularly in pharmacotherapy. Illicit drug use remains pervasive, with 70.5 million individuals (24.9%) reporting use of substances such as marijuana, cocaine, heroin, methamphetamine, and hallucinogens, alongside misuse of prescription drugs (Hee 2018). These statistics underscore the widespread impact of SUD on public health and the pressing need for innovative interventions that address its complex pathology. Compounding this crisis, SUD frequently co‐occurs with mental health conditions, as 22.8% of adults with any mental illness and 5.7% with serious mental illness in 2023 also faced SUD (SAMHSA 2023). The economic toll is immense, with societal costs surpassing $400 billion annually due to healthcare expenses, lost productivity, and criminal justice involvement (Fardone et al. 2023). The opioid epidemic alone has significantly exacerbated these issues, evidenced by a record 93,000 overdose deaths in 2020—a 29.4% increase from 2019 (Blanco et al. 2020) —and a continued rise in opioid use between 2021 and 2023. Recent data emphasize the urgency of addressing SUD. In 2023, 48.5 million individuals (17.2% of the U.S. population) were diagnosed with SUD. Among these, 27.2 million (9.6%) experienced drug use disorder (DUD), including 5.7 million (2%) with OUD. Additionally, AUD impacted 28.9 million people (10.2%), while tobacco use disorder (TUD) affected 38.7 million (13.7%).

This review delves into the current state of pharmacotherapy for SUD, analyzing treatment modalities across its major subtypes and exploring emerging innovations such as nanomedicine‐based therapies and personalized medicine approaches. Additionally, the review underscores recent advancements in long‐acting formulations, novel therapeutic targets, and integrated pharmacological, behavioral, and device‐based interventions. Recognizing SUD as a chronic, relapsing condition necessitates a paradigm shift toward nano‐inspired pharmacotherapies and long‐term management strategies that address the holistic aspects such as the biological, psychological, and social dimensions of addiction (Theodorakis et al. 2024). By evaluating neurobiological mechanisms, pharmacological treatments, and psychosocial dynamics, this analysis aims to illuminate pathways for the next generation of pharmacotherapies for SUDs. It advocates for future research and policy reforms that enhance the integration of nanomedicine‐inspired pharmacotherapy with complementary interventions, ultimately alleviating the substantial burden of addiction on individuals and society. Special emphasis is placed on the transformative potential of nanomedicine in addressing existing gaps, paving the way for more effective and equitable care models.

## 2 Current Landscape of Pharmacotherapy for SUD

Pharmacotherapy has become a cornerstone of SUD management, offering evidence‐based strategies to reduce morbidity and mortality. For instance, methadone, buprenorphine, and naltrexone have demonstrated efficacy in treating OUD, while naltrexone, acamprosate, and disulfiram play crucial roles in managing AUD (Table 1).

**TABLE 1:** Currently available FDA‐approved therapies for opioid and alcohol use disorders.

Medications/Mechanism	Therapeutic efficacy	Remarks
Methadone: A full μ‐opioid receptor agonist	Reduces withdrawal symptoms and cravings without inducing euphoria	Reduce all‐cause mortality (Chona 2017; Wang 2019)
Buprenorphine: a partial μ‐opioid receptor agonist	Offers a safer profile due to its ceiling effect on respiratory depression, lowering overdose risks, reduce all‐cause mortality	Sublingual tablets, buccal films, long‐acting injectables (Pande and Piper 2020)
Naltrexone: an opioid antagonist, prevents euphoric effects by blocking opioid receptors	Moderate efficacy, effective in promoting abstinence and reducing opioid cravings	Extended‐release (monthly injectable format) (Rozenberg 2020)
Naltrexone: reduces the reinforcing effects of alcohol consumption	Reduced the risk of heavy drinking by 83% versus placebo	Available in both oral and injectable forms) (Collins et al. 2021; Lobmaier et al. 2011)
Acamprosate: targets glutamate and GABA neurotransmission.	Reducing cravings and withdrawal symptoms	Restores neurochemical balance disrupted by chronic alcohol use (Liang and Olsen 2014)
Disulfiram: Operates as a deterrent	Effective in promoting abstinence by discouraging alcohol consumption in individuals motivated to maintain sobriety.	Causes unpleasant physiological reactions when alcohol is consumed (De Sousa 2019)

However, significant gaps in access, adherence, and utilization persist and the challenges within SUD treatment vary significantly across different substance types. OUD remains among the most severe, with high morbidity and mortality rates. Although effective pharmacotherapies exist, their accessibility remains a challenge, especially in rural and under‐resourced communities. Integrating behavioral strategies with pharmacotherapy is crucial for improving adherence and patient outcomes. For instance, a 2019 study reported that fewer than 20% of individuals with OUD received Medication‐Assisted Treatment (MAT), highlighting systemic barriers such as stigma, regulatory hurdles, and the lack of specialized providers (National Academies of Sciences, Engineering, and Medicine 2018). Geographic disparities further exacerbate these issues, with rural areas particularly affected. Policy changes, including the elimination of waiver requirements for treating small patient cohorts, represent critical steps toward addressing these challenges (Richman et al. 2019).

Other SUDs such as the stimulant use disorders, involving substances such as methamphetamine, cocaine, and chronic cannabis use, have increased substantially, placing additional strain on healthcare systems (Fischer et al. 2021). However, unlike OUD and AUD, no FDA‐approved medications exist for stimulant drug use disorders (DUD) and chronic cannabis use disorders (CUD) creating a critical treatment gap (Lee et al. 2024), (Brezing and Levin 2018). Emerging approaches, such as dopamine modulation and transcranial magnetic stimulation, are under investigation to address this unmet need. This gap is concerning given the rising prevalence of stimulant misuse and its associated health and societal burdens (Hood et al. 2020). Emerging treatments are being explored, including combinations of extended‐release naltrexone and bupropion, which demonstrated significantly higher response rates for this combination compared to placebo (Grilo et al. 2021). Additionally, dopamine modulators like modafinil and methylphenidate have shown promise in reducing cocaine use, particularly in individuals with comorbid conditions such as attention‐deficit hyperactivity disorder (Brandt et al. 2021). Preclinical research into innovative strategies such as vaccines targeting cocaine and methamphetamine offers further hope for addressing this critical treatment gap. Similarly, for CUD, emerging therapies are under investigation, including cannabinoid receptor partial agonists such as nabiximols, which have shown efficacy in reducing cannabis use and alleviating withdrawal symptoms (Alayoubi et al. 2024). Additionally, repurposed medications like gabapentin have demonstrated potential in small pilot studies. Researchers are also exploring the modulation of the endocannabinoid system using fatty acid amide hydrolase inhibitors to address cravings and withdrawal (Best 2022). Table 2 provides a summary of selected clinical trials focusing on novel pharmacotherapies for SUDs, highlighting their potential to reshape treatment paradigms.

**TABLE 2:** A list of selected clinical trials focusing on novel pharmacotherapies for SUDs.

Title (clinical trial id)	Pharmacotherapy approach	Key findings	Implications
Naltrexone for OUD with Pharmacogenetic Focus (NCT03226223)	Genetic‐targeted treatment	Highlights personalized medicine pathways for optimizing OUD treatments.	Guides precision‐based pharmacotherapy development.
Cocaine Dependence and PPARγ Agonist (NCT02774343)	Pioglitazone (Anti‐inflammatory)	Supports anti‐inflammatory therapies for cocaine dependence.	Expands options for addressing neuroinflammation.
D‐Cycloserine in Cocaine Cue Exposure (NCT00780442)	NMDA receptor modulation	Demonstrates efficacy in reducing craving through receptor‐targeted therapy.	Highlights potential for cue‐specific craving reduction.

## 3 Nanomedicine Innovations in Pharmacotherapy Treatment for SUD

### 3.1 Progress of Nanomedicine Approach for Therapeutics

**FIGURE 1:**
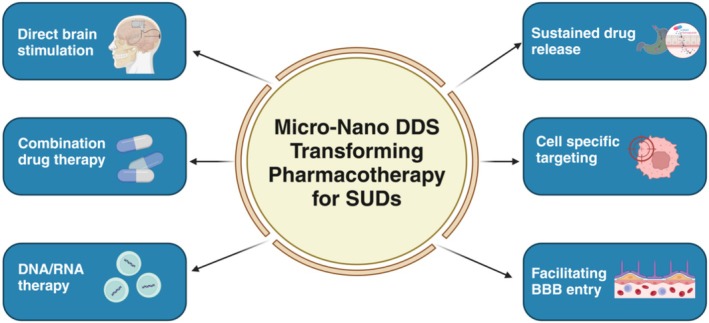
A cartoon describing various micro‐nano drug delivery systems (DDS) approaches which can significantly advance pharmacotherapy for SUDs.

Nanotechnology in healthcare, widely referred to as nanomedicine, has ushered in a paradigm shift by offering more precise, efficient, and personalized methodologies for disease prevention and treatment. Over the last two decades, advancements in nanotechnology have rapidly evolved, enabling innovations that address limitations of conventional medical interventions. For instance, the discovery of novel nano‐biomaterials, nanoscale‐inspired multifunctional drug delivery systems, novel imaging systems, advanced biosensors, and implantable devices now provide solutions for diseases that previously lacked effective treatment options (Kar et al. 2022; Markoutsa et al. 2021; Mohapatra et al. 2013). The various benefits of utilizing nanomedicine, especially developing the micro‐nano drug delivery systems (DDS) are highlighted in Figure 1. For example, lipid‐based nanoparticles (LNPs), which have become versatile carriers for various drugs, including small molecules and biologics, enhance drug stability, solubility, and bioavailability while ensuring controlled‐release kinetics, thereby minimizing side effects (Mehta et al. 2023) and improving therapeutic efficacy by over 40%, particularly in conditions requiring precision targeting (Jung et al. 2022). Furthermore, non‐invasive therapeutic options such as nanoshell‐based photothermal therapies offer a safer alternative to traditional surgical interventions for brain‐related conditions (Skandalakis et al. 2020). Additionally, nanotechnology‐based coatings, such as those using materials like gold, titanium dioxide, and zinc oxide, are frequently employed in medical devices to enhance biocompatibility, durability, and efficiency (Ramasamy and Lee 2016). Emerging innovations such as ligand‐functionalized nanoparticles and biomimetic nanocarriers are further enhancing drug targeting specificity, particularly in addiction‐related pathways (Han et al. 2024). Additionally, the field of regenerative medicine and personalized cancer treatment has greatly benefited from nanofiber scaffolds composed of biocompatible polymers (Wang et al. 2020). These scaffolds provide a three‐dimensional microenvironment that facilitates cell adhesion, proliferation, and differentiation, leading to breakthroughs in tissue engineering and organ transplantation.

### 3.2 Nanomedicine‐Inspired Therapies for SUDs—Preclinical Studies

Nanomedicine has fundamentally transformed the treatment landscape for SUD by addressing several longstanding challenges. These include the efficient delivery of therapeutic agents to the brain, achieving sustained therapeutic efficacy, and minimizing systemic toxicity (Kasina et al. 2022). For instance, nanofiber scaffolds have shown a 60% improvement in functional recovery rates in degenerative disease models, highlighting their potential to restore tissue functionality in SUD patients who experience neurodegeneration due to chronic substance misuse (Han et al. 2020). As a highly interdisciplinary field, nanomedicine harnesses the distinctive characteristics of nanoscale materials to craft therapies that align with the neurobiological complexities of addiction, focusing on precise drug delivery, overcoming the BBB, and reducing systemic toxicity for improved treatment outcomes (Patra et al. 2018). Nanoparticles are now being engineered with BBB‐penetrating capabilities using transferrin, lactoferrin, and glucose transporter ligands to enhance drug transport into the central nervous system (CNS) (Ding et al. 2020). Recent advancements in artificial intelligence (AI)‐driven drug discovery have further complemented nanomedicine‐based approaches, enabling the identification of precise molecular targets and innovative therapeutic strategies, which have shown promise in overcoming the limitations of conventional pharmacotherapies (Patne et al. 2024; Paul et al. 2021).

Since its emergence in the late 1990s, nanotechnology has facilitated the development of innovative drug delivery systems, such as lipid nanoparticles, gold nanoparticles, carbon nanotubes, magnetic nanoparticles, silica nanoparticles, dendrimers, and polymeric nanoparticles (Chuang et al. 2024; Patra et al. 2018). These platforms have demonstrated key advantages, including enhanced permeability across the BBB, reduced systemic toxicity, and sustained therapeutic effects, which are essential in overcoming the challenges of targeting addiction‐relevant brain regions. Nanocarriers capable of active and passive transport mechanisms are now being explored to enhance brain penetration (Ahlawat et al. 2020). Additionally, exosome‐based drug delivery and bioengineered nanoparticles are gaining attention for their ability to mimic endogenous transport mechanisms, increasing therapeutic retention in addiction‐related brain circuits (Lopes et al. 2023).

Recent advances in drug delivery systems targeting the BBB have shown promising potential for treating neurological diseases. For example, NPs functionalized with ligands that bind to specific receptors on the BBB facilitate the transport of therapeutic agents across the barrier (Wang et al. 2023). Furthermore, lipid‐based or polymeric NPs, including dendrimers, are being developed for both systemic and intranasal administration to bypass the BBB and directly deliver neurotherapeutics (Das, Mayilsamy, et al. 2019; Das, Tang, et al. 2019; Mayilsamy et al. 2020). By attaching brain‐targeting ligands, such as peptides, proteins, or antibodies, these nanovesicles can enhance the precision of drug delivery to specific brain cells (Moreira et al. 2024). Another advance involves systems using biomimetic membranes and exosomes, which mimic natural biological processes to improve drug delivery efficiency. Exosomes are naturally occurring vesicles that can be engineered to carry drugs across the BBB (Wang et al. 2022). Advances in nanotechnology have enabled the development of NPs that can release drugs in a controlled manner, improving the pharmacokinetic behavior of drugs and reducing withdrawal symptoms and relapse risk (Alghamdi et al. 2022). Nanocarriers designed for neuroprotection and neuroregeneration are now being explored, aiming to mitigate brain damage caused by chronic substance use (Shabani et al. 2023). Functionalized nanoparticles delivering neurotrophic factors (e.g., BDNF, GDNF) have demonstrated potential in reversing neuronal loss in preclinical models (Bondarenko and Saarma 2021).

**FIGURE 2:**
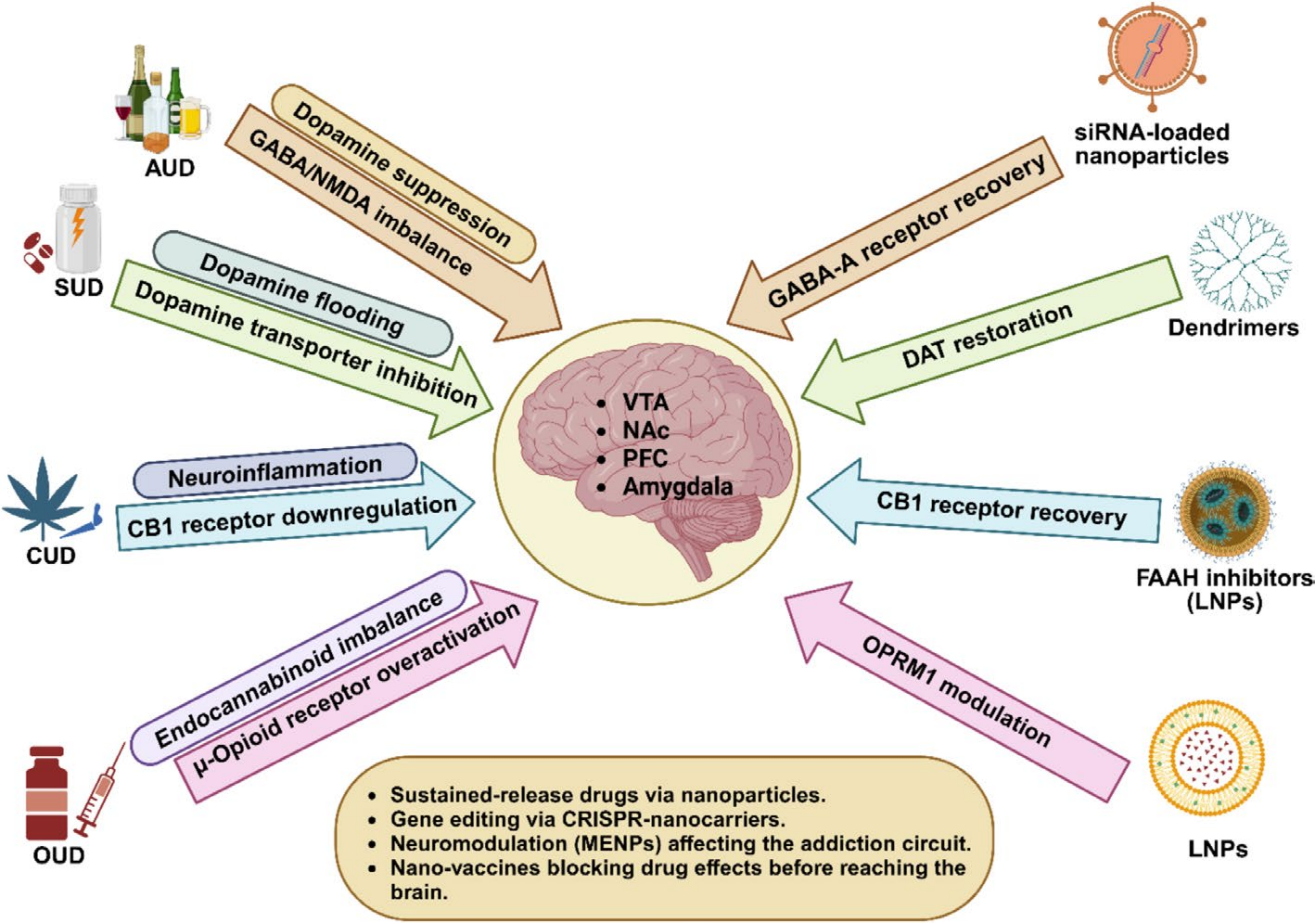
Neurobiological mechanisms of substance use disorders (SUDs) and nanomedicine‐driven interventions.

These innovations are paving the way for more effective treatments for conditions like traumatic brain injury, Alzheimer's disease, Parkinson's disease, and glioblastoma by overcoming the challenges posed by the BBB (Das et al. 2012). Similarly, in the context of SUD, nanomedicine is now integrating optogenetic modulation, where nanoparticles are used to deliver light‐sensitive proteins, allowing precise control over addiction‐related neural circuits (All et al. 2019; Stuber and Mason 2013). This novel approach could revolutionize treatment strategies by modulating reward pathway activity in real‐time. The neurobiological mechanisms underlying SUDs vary depending on the substance of abuse, with AUD associated with GABA/NMDA imbalance, SUD involving dopamine dysregulation, CUD linked to CB1 receptor downregulation, and OUD characterized by μ‐opioid receptor overactivation (Figure 2). Nanomedicine‐driven interventions, such as siRNA‐loaded nanoparticles, dendrimers, FAAH inhibitors, and LNPs, are designed to restore receptor function, modulate addiction circuits, and reduce neuroinflammation. These therapies leverage the precision of nanotechnology to enable targeted drug delivery, sustained therapeutic effects, and modulation of addiction‐related pathways in the brain.

**FIGURE 3:**
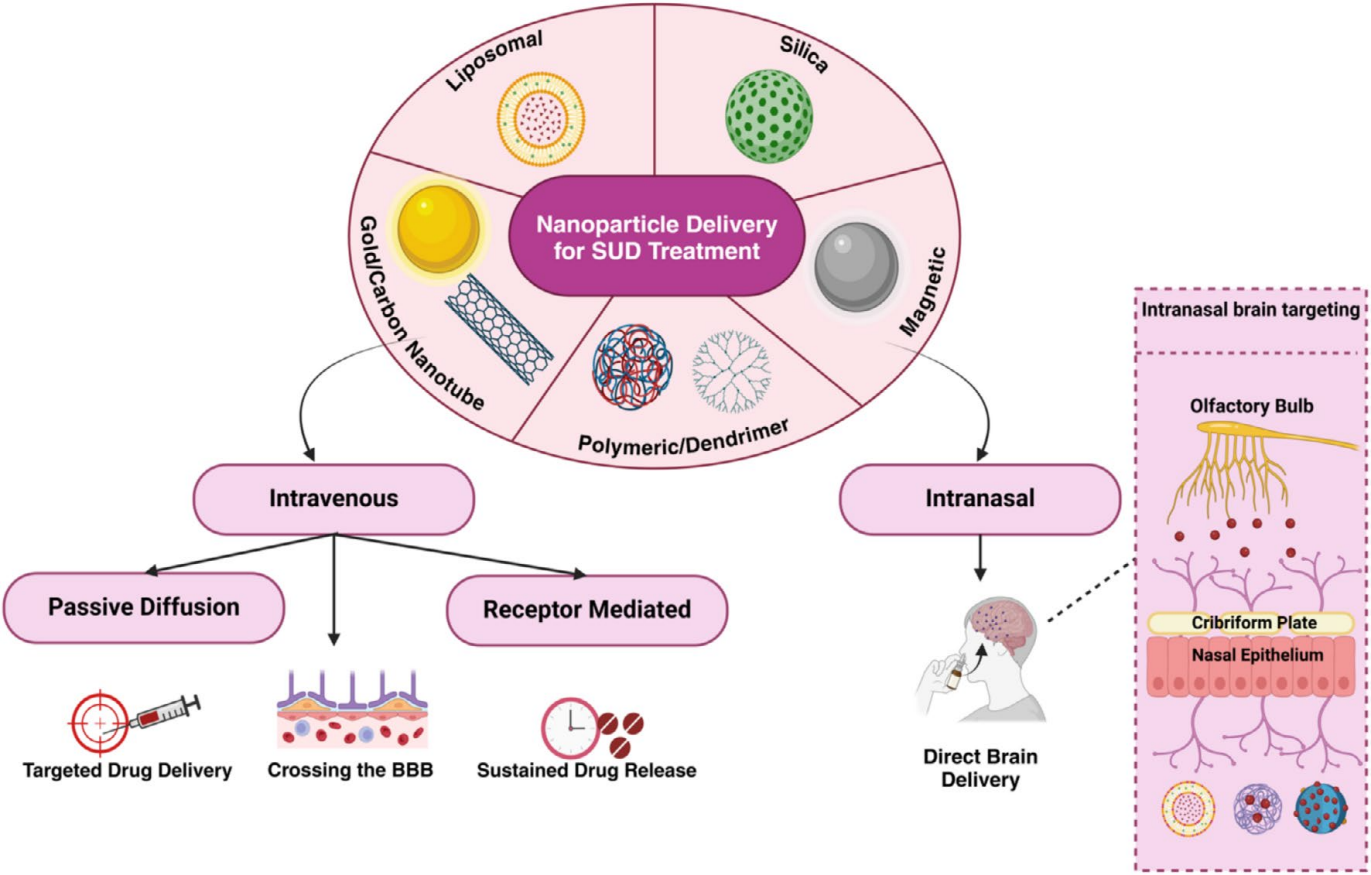
Nanoparticle delivery mechanisms for substance use disorder (SUD) treatment.

NPs not only facilitate the transport of drugs across the BBB but also enable selective targeting of addiction‐related brain regions, such as the mesolimbic reward pathway, where dopaminergic signaling plays a central role in drug‐seeking behaviors (Hersh et al. 2022). Figure 3 provides a comprehensive overview of NP types and their delivery pathways, including receptor‐mediated, intranasal, and passive diffusion mechanisms. It highlights how NPs—such as liposomal, polymeric, magnetic, silica, and gold nanoparticles—overcome the BBB and deliver therapeutic agents precisely to targeted brain regions. This visual representation underscores their transformative role in addressing the pharmacological barriers to effective SUD treatment while minimizing side effects and enhancing sustained drug release.

Thus, targeted nanomedicine revolutionizes addiction treatment by addressing the limitations of conventional pharmacotherapies, particularly in overcoming the challenges of the BBB (Desai 2012). Advanced nanomedicine platforms facilitate the precise delivery of therapeutic agents to addiction‐relevant brain regions, significantly enhancing specificity and efficacy (Gong 2020). Future directions should focus on optimizing nanoparticle properties such as size, charge, and ligand modification to enhance selectivity for addiction‐relevant brain circuits. Additionally, multifunctional nanocarriers integrating both therapeutic and diagnostic capabilities (theranostics) hold promise for personalized SUD treatment approaches. Artificial intelligence (AI)‐guided drug delivery and machine learning algorithms are also being leveraged to predict the optimal nanoparticle design for improving treatment outcomes in SUDs (Mazumdar et al. 2025).

#### 3.2.1 Micro‐Nano Advancing Drug Delivery Systems Through Nanotechnology

One of the most significant challenges in treating SUDs is the efficient delivery of therapeutic agents to the CNS. The BBB, while serving as a vital protective mechanism, significantly limits the entry of pharmacological treatments into addiction‐relevant neuronal circuits (Daneman and Prat 2015). Nanoparticles have emerged as transformative solutions, leveraging their diverse properties to overcome this barrier and enable targeted drug delivery to specific brain regions, thereby enhancing the precision and efficacy of SUD treatment (Gude 2024). Further, nanocarrier systems like liposomes, micelles, and dendrimers are being used to deliver neuroprotective agents that can help repair and protect brain cells affected by substance abuse (Naqvi et al. 2020). Table 3 provides selected examples of nano‐DDS and provides a comparative overview of nanoparticle types, detailing their advantages, disadvantages, alternate approaches and their potential therapeutic applications in SUD management. These data underscore the transformative potential of nanomedicine in addressing critical gaps in addiction treatment while highlighting areas that require further research and optimization.

**TABLE 3:** Preclinical studies of nano‐systems for potential anti‐SUD therapies.

Nano‐system (NS)	Anti‐SUD PTx (Route)	Outcome	Positives/negatives and alternate approach (AA)	References
Carbon nanotubes (CNT)	Methampheta‐mine (ICV)	Significant inhibition of METH addiction and craving through dopamine oxidation	**Pos**: Strong drug binding, high surface area	(Xue et al. 2016)
			**Neg**: Poor biodegradability	
			**AA**: Use biocompatible CNT with less toxicity	
Dendrimers (Polymeric)	Cocaine Vaccine (IM)	Elicited strong cocaine‐specific antibody response and reduced rewarding effects of cocaine	**Pos**: Precision targeting and low toxicity	(Lowell et al. 2020)
			**Neg**: Complex dendrimer synthesis	
			**AA**: G4 dendrimer synthesis has been optimized in new‐generation dendrimers	
Gold Nanoparticles	siRNA targeting DARPP‐32 (IV)	Modulated dopaminergic signaling and opioid symptom withdrawal	**Pos**: Crosses BBB efficiently for gene therapy	(Bonoiu et al. 2009)
			**Neg**: Gold particle accumulation and potential cytotoxicity	
			**AA**: Replace with biocompatible polymeric particles	
Lipid‐based Nanoparticles	Nicotine Vaccine (SQ), Cocaine Vaccine (IM)	Reduced substance levels and elicited strong vaccine‐induced immune response	**Pos**: Slow release and BBB penetration	(Brisse et al. 2020)
			**Neg**: Low uptake efficiency	
			**AA**: Use ligand‐targeted nanoparticles	
Polymeric1 Nanoparticles (PNPs)	Naloxone (SQ), Buprenorphine (IN), GDNF (IC)	Reduced withdrawal symptoms and enhanced drug efficacy	**Pos**: High biocompatibility, surface modification options	(Curtis et al. 2017)
			**Neg**: Self‐aggregation	
			**AA**: Alter surface chemistry to reduce aggregation	
Silica Nanoparticles	Methamphetamine, Naltrexone, Methadone (IV)	Reduced neurotoxicity in methamphetamine and methadone‐induced withdrawal	**Pos**: High surface area and stability	(Moradi et al. 2024)
			**Neg**: Aggregation and immunogenicity	
			**AA**: Use biocompatible coatings	

Abbreviations: AA, alternate approach; GDNF, glial cell line‐derived neurotrophic factor; ICV, intracerebroventricular delivery; IM, intramuscular administration; IN, intranasal administration; IV, intravenous delivery; NS, nanosystem; PTx, pharmacotherapeutics; SQ, subcutaneous administration; SUD, gubstance use disorder; Vac, vaccine.

Lipid nanoparticles (LNPs) have gained widespread attention for their ability to encapsulate both hydrophilic and hydrophobic drugs. By stabilizing therapeutic agents and protecting them from premature degradation, LNPs improve drug bioavailability and systemic stability. Recent studies have demonstrated that LNPs can enhance brain drug delivery by approximately 30% compared to traditional formulations (Costa et al. 2021). This advancement is particularly critical for disorders like tobacco use disorder, where LNPs used in nicotine vaccines have shown up to a 40% improvement in treatment outcomes in preclinical models. Their versatility and biocompatibility position LNPs as a cornerstone in SUD pharmacotherapy, particularly through receptor‐mediated pathways. Moreover, lipid‐based nanoparticles have been shown to enhance drug retention in the brain, increasing therapeutic bioavailability by approximately 30% compared to conventional formulations (Fernandes et al. 2021). These nanoparticles leverage their biocompatible lipid bilayers to encapsulate drugs and prevent rapid degradation in systemic circulation. As a result, LNPs not only improve the pharmacokinetics of therapeutic agents but also reduce dosing frequency, thereby addressing adherence challenges commonly observed in SUD patients. For example, nicotine and cocaine vaccines formulated with LNPs have exhibited significantly higher efficacy in preclinical trials, demonstrating their potential for clinical translation (Vavilis et al. 2023).

##### 3.2.1.1 LNP‐Enabled Neutralization for SUDs

LNP‐based‐Neutralization represent a promising approach in SUD treatment, particularly for nicotine and cocaine addiction. These approaches work by encapsulating drug‐specific antigens, ensuring enhanced stability, sustained release, and improved immune response activation (Shorter and Kosten 2011). Unlike traditional approaches that often suffer from rapid antigen degradation, LNPs provide a protective environment that enhances antigen bioavailability and prolongs immune response.

Preclinical studies have demonstrated that LNP‐encapsulated nicotine ‐neutralizations lead to reduced nicotine levels in the bloodstream by triggering a strong antibody response that prevents the drug from crossing the BBB and reaching the brain (Fahim et al. 2011). Similarly, cocaine neutralizing agents formulated with LNPs have shown up to 40% efficacy in blocking cocaine‐induced reinforcement behaviors, significantly reducing relapse rates in preclinical models (Carrera et al. 2000).

**FIGURE 4:**
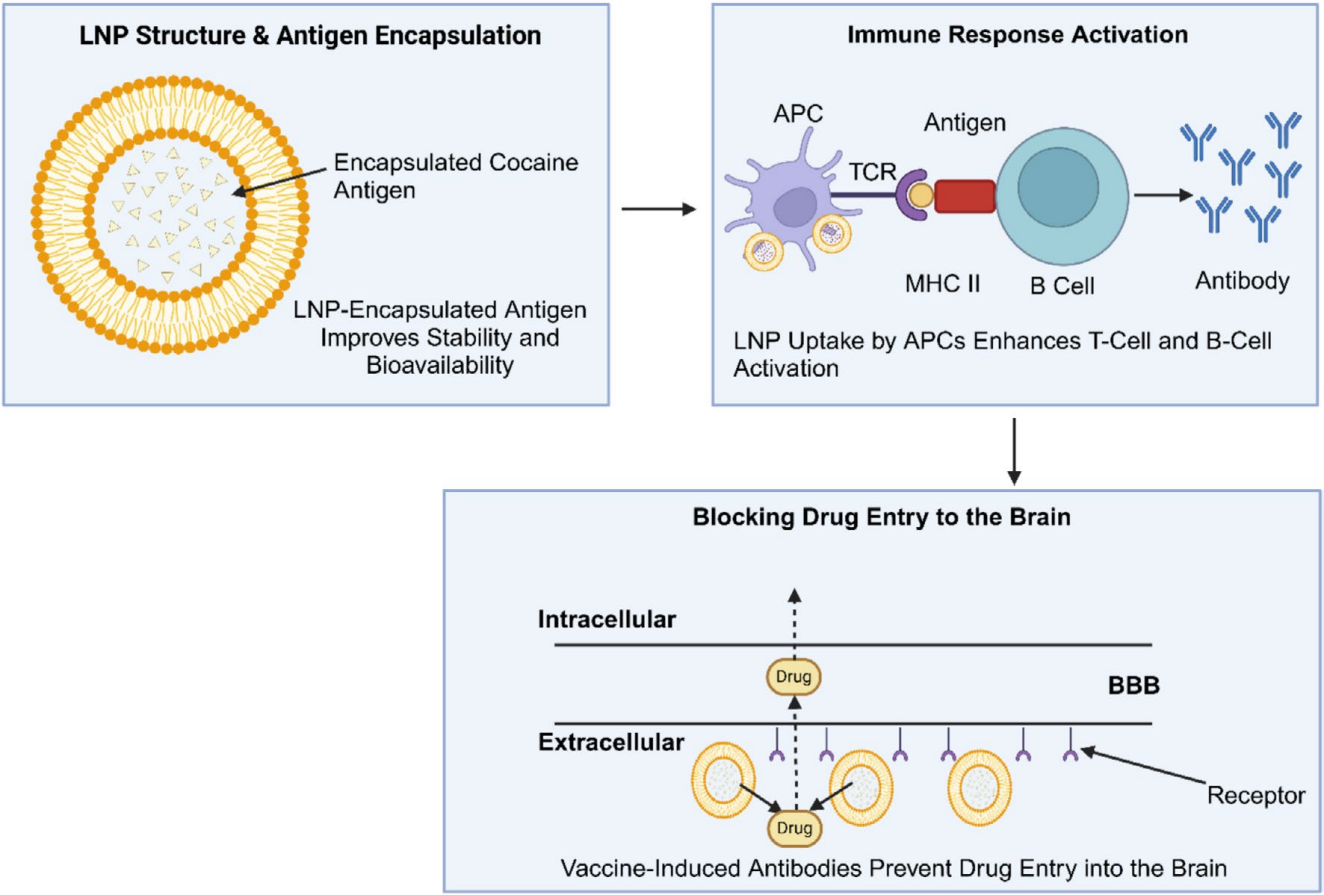
Mechanism of LNP‐based neutralizations for substance use disorders (SUDs).

Despite these promising findings, challenges remain in optimizing LNP‐based neutralizations. Low uptake efficiency of antigens by immune cells can limit potency, necessitating modifications in surface ligands or adjuvant formulations to enhance immunogenicity (Reed et al. 2013). Additionally, potential immune system overstimulation and long‐term safety concerns require further investigation through extended preclinical and clinical trials. When compared to traditional approaches, LNP‐based neutralizations offer several key advantages, including improved antigen stability, prolonged immune activation, and targeted antigen delivery. Conventional vaccines often require multiple booster doses due to rapid antigen clearance, whereas LNP formulations provide extended antigen presentation, reducing the need for frequent dosing and improving patient compliance (Bhardwaj et al. 2020). The ability of LNPs to facilitate controlled immune activation makes them a promising innovation for preventing substance use relapse and addiction recurrence. Figure 4 illustrates the mechanism of LNP‐based neutralizations for SUDs. The diagram demonstrates the structural composition of LNPs encapsulating drug‐specific antigens, which enhance antigen stability and bioavailability. It also depicts the immune response activation, where LNP uptake by antigen‐presenting cells (APCs) stimulates T‐cell and B‐cell activation, leading to antibody production. Furthermore, the figure highlights how antibodies prevent drug molecules from crossing the blood–brain barrier (BBB), thereby blocking drug entry into the brain and mitigating substance use relapse.

Magnetic nanoparticles (MNPs) offer another innovative approach by utilizing external magnetic fields to guide drug delivery. These nanoparticles are engineered with magnetic cores that enable precise localization of therapeutic agents within the brain (Roet et al. 2019). For instance, MNPs have been successfully directed to the nucleus accumbens, a critical region in the reward pathway implicated in addiction (Cooper et al. 2017). This targeted delivery mechanism not only improves therapeutic efficacy but also minimizes off‐target effects, reducing systemic side effects. Preclinical studies highlight a 50% improvement in drug efficacy when MNPs are employed for treating opioid and stimulant use disorders, emphasizing their potential for addressing relapse and withdrawal symptoms (Olaitan et al. 2024). Intranasal administration of MNPs has further streamlined their application, bypasses the BBB and directly targeting brain regions. Preclinical studies have been instrumental in demonstrating the efficacy of these nanoscale technologies. MNPs, for instance, have been engineered for precise drug delivery to addiction‐relevant brain regions under the influence of external magnetic fields. This targeted approach minimizes off‐target effects and systemic side effects, improving drug efficacy by nearly 50% in preclinical addiction models (Tian et al. 2024). MNPs also offer the added advantage of controlled drug release, allowing therapeutic agents to maintain efficacy over an extended period, which is particularly beneficial for reducing relapse rates in opioid and stimulant addiction models (Sagar 2013).

Recent advancements in magnetoelectric nanoparticles (MENPs) have highlighted their potential in modulating neural circuits implicated in addiction. These nanoparticles combine drug delivery with brain stimulation techniques, such as modulating dopaminergic signaling, to reduce cravings and relapse behaviors. Preclinical models using MENPs demonstrated a 40% reduction in relapse rates compared to control groups (Volkow et al. 2019). Additionally, MENPs' ability to cross the BBB and deliver therapeutic payloads to addiction‐relevant regions further emphasizes their transformative potential in addiction medicine (Hersh et al. 2016). However, despite their promise, the scalability and long‐term safety of MENPs remain key challenges. Addressing these limitations through comprehensive clinical trials is crucial for translating these innovations into viable treatments for SUD.

Polymeric nanoparticles (PNPs) extend nanotechnology's capabilities by offering sustained drug‐release properties, ensuring consistent therapeutic levels over time. These nanoparticles are particularly effective in managing chronic conditions like opioid and alcohol use disorders, where frequent dosing can hinder patient adherence (Hawthorne et al. 2022). Studies on polymeric nanoparticles delivering buprenorphine have demonstrated a 35% reduction in relapse rates compared to traditional treatments, underscoring their value in long‐term management strategies for SUDs (Shulman et al. 2019). These nanoparticles primarily leverage passive diffusion mechanisms for delivering drugs across biological barriers.

Silica nanoparticles (SNPs) and gold nanoparticles (AuNPs) further enhance the toolkit of nanotechnology for SUD treatment. Silica nanoparticles, with their large surface area and chemical stability, enable high drug‐loading capacity and precision delivery through receptor‐mediated transport. On the other hand, gold nanoparticles, due to their small size and tunable properties, effectively utilize passive diffusion to cross cellular membranes and deliver drugs directly to addiction‐relevant brain areas (Moreira et al. 2024). Both systems represent innovative approaches to improving treatment outcomes while minimizing systemic toxicity.

Silica nanoparticles, with their large surface area and chemical stability, have emerged as promising platforms for the delivery of drugs like naltrexone and methadone, particularly for managing opioid use disorders. However, challenges such as aggregation and immunogenicity remain to be resolved before clinical implementation (Abeer et al. 2020).

### 3.3 Tailored Nanomedicine Approaches for SUD

The concept of tailoring nanomedicine for SUD treatment is rooted in pharmacogenomics, which highlights interindividual variability in responses to traditional therapies. For instance, variations in the OPRM1 gene encoding the μ‐opioid receptor have been linked to differential responses to opioid antagonists like naltrexone (Taqi et al. 2019). A recent study reported that patients with specific OPRM1 alleles experienced a 25% greater reduction in opioid cravings when treated with naltrexone delivered through LNPs (Alam and Singh 2023).

Nanomedicine's precision targeting capabilities enable enhanced drug delivery to addiction‐relevant molecular pathways, reducing side effects and improving treatment adherence. For example, polymeric nanoparticles have been shown to provide sustained drug release over 72 h, ensuring consistent therapeutic levels in the brain and significantly reducing withdrawal symptoms in preclinical opioid addiction models (Brigham et al. 2021; Kasina et al. 2022).

### 3.4 Cell and Tissue‐Specific Targeting for SUDs

**FIGURE 5:**
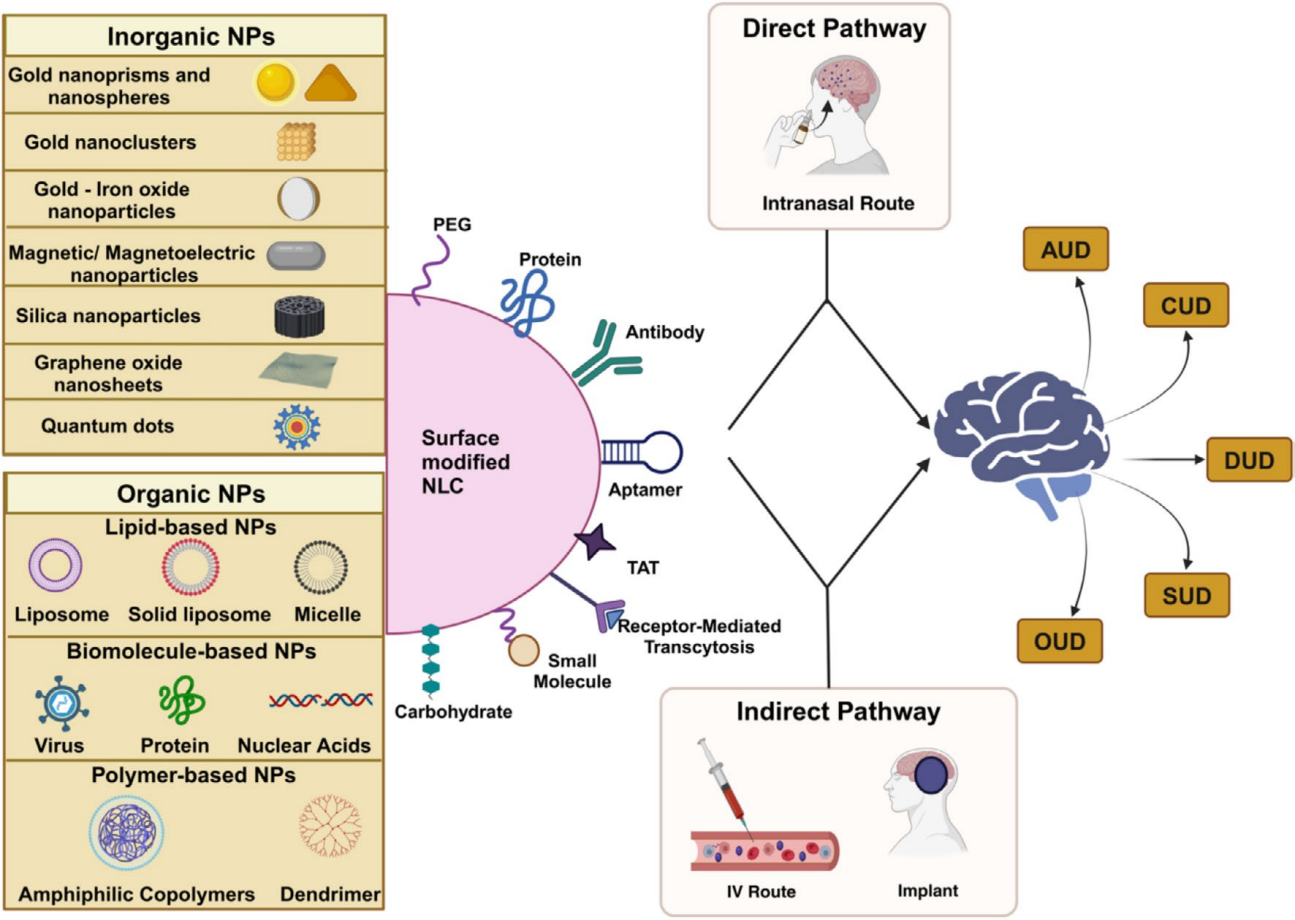
Schematic representation of nanoparticle‐based targeted drug delivery for SUD treatment.

These nanoparticles are engineered to cross the BBB efficiently, utilizing biocompatible coatings and ligands that facilitate receptor‐mediated transcytosis. For example, lipid nanoparticles (LNPs) modified with transferrin receptors have demonstrated an 80% increase in delivery to the mesolimbic reward pathway, a critical region implicated in addiction (Dunigan and Roseberry 2022). This targeted approach enhances treatment efficacy and reduces systemic side effects. This targeted approach enhances treatment efficacy and reduces systemic side effects (Figure 5).

This targeted approach can enhance the effectiveness of treatments and reduce side effects (Naqvi et al. 2020). Nanoparticles can be administered through both direct and indirect pathways, including intravenous (IV) injection, implantable devices, and intranasal (IN) delivery. Indirect pathways (IV and implant) allow systemic or localized sustained release, while direct pathways (IN) enable nose‐to‐brain transport via the olfactory and trigeminal nerve routes.

Nanoparticles can be engineered to target specific neuronal subtypes within addiction‐relevant brain circuits, offering unparalleled precision in drug delivery. For example, dopaminergic neurons in the mesolimbic reward pathway play a central role in the reinforcing effects of addictive substances (Adinoff 2004). Surface‐functionalized nanoparticles, such as those modified with ligands or antibodies, enable selective cellular uptake by targeting specific receptors expressed on addiction‐relevant neuronal populations (Zhang et al. 2021). Targeting these neurons with nanoparticle‐based therapies allows for the modulation of dopamine signaling pathways, thereby reducing drug‐seeking behaviors. Studies have reported that surface‐modified nanoparticles, compared to unmodified formulations, achieve a 40% improvement in therapeutic efficacy (Cheng et al. 2023). Emerging research explores the potential of optogenetics in addiction modulation by integrating nanoparticles for light‐sensitive neural circuit control. Nanoparticles can be engineered to deliver optogenetic actuators, such as channel rhodopsin, into addiction‐related brain regions, allowing for precise light‐activated control over dopaminergic signaling (Baker 2023). This approach has shown promise in preclinical models, enabling real‐time manipulation of neuronal activity to reduce drug‐seeking behaviors.

For instance, nanomedicine approaches focused on modulating the dopaminergic communication pathways in the brain—crucial in the development of addiction—offer significant promise (Giménez et al. 2024). Surface‐functionalized nanoparticles, such as those modified with ligands or antibodies, have shown remarkable success in achieving selective cellular uptake. These modifications enhance the nanoparticles' ability to bind to specific receptors expressed on addiction‐relevant neuronal populations, ensuring that therapeutic agents are delivered precisely where they are needed (Chehelgerdi et al. 2023). Studies have reported a 40% improvement in therapeutic efficacy with surface‐modified nanoparticles compared to unmodified formulations (Yetisgin et al. 2020). For instance, nanomedicine approaches focused on modulating the dopaminergic communication pathways in the brain, which are crucial in the development of addiction (Tyagi and Pandey 2016). Surface‐functionalized nanoparticles offer further precision through cell‐specific targeting. For instance, nanoparticles functionalized with ligands targeting dopaminergic neurons in the mesolimbic pathway enable selective modulation of dopamine signaling, reducing drug‐seeking behaviors. Such technologies have achieved a 35% improvement in treatment outcomes compared to non‐targeted systems (Baik 2013). The MENPs are being investigated for their potential in neural circuit modulation. By utilizing externally controlled magnetic fields, MENPs can non‐invasively regulate neuronal activity, providing an alternative to traditional optogenetic techniques (Giménez et al. 2024). This innovation has been explored as a strategy to enhance the precision of SUD treatment by enabling spatially and temporally controlled neuromodulation.

Another innovative approach is the use of magnetic nanoparticles (MNPs) for precise drug localization. MNPs, guided by external magnetic fields, have been shown to deliver dopamine modulators and opioid receptor antagonists directly to the nucleus accumbent (Hauser et al. 2015). Preclinical models indicate a 50% improvement in drug efficacy and a 40% reduction in relapse rates with MNP‐based therapies for opioid and stimulant use disorders (Yahyavi‐Firouz‐Abadi and See 2009). These targeted approaches further minimize off‐target effects and reduce cravings and withdrawal symptoms, paving the way for more effective and precise treatments for SUDs.

### 3.5 Nano‐Enabled Sustained Release and Prolonged Therapeutic Effect

In addition to cell‐specific targeting, polymeric nanoparticles with sustained‐release properties offer a prolonged therapeutic effect and enhancing patient adherence. By maintaining steady drug concentrations in the brain, these nanoparticles reduce the need for frequent dosing, a factor that has been shown to significantly improve adherence rates among SUD patients (Kasina et al. 2022). For example, preclinical models of opioid use disorder treated with polymeric nanoparticles delivering buprenorphine demonstrated a 35% reduction in relapse rates compared to conventional treatment protocols (Ronquest et al. 2018). Polymeric nanoparticles capable of releasing therapeutic agents over 72 h have demonstrated a 35% reduction in relapse rates in opioid addiction models (Heyns et al. 2024). Recent research has highlighted the role of neuroinflammation in SUD withdrawal symptoms, where chronic substance use leads to microglial activation and increased inflammatory cytokine production (e.g., IL‐6, TNF‐α, IL‐1β) (Wei et al. 2020). This inflammatory response exacerbates withdrawal symptoms such as anxiety, depression, and drug cravings, making relapse more likely. Nanoparticle‐based drug delivery systems offer a promising solution by enabling targeted anti‐inflammatory therapies. Polymeric nanoparticles, lipid‐based nanocarriers, and exosome‐mimetic vesicles can encapsulate and deliver anti‐inflammatory agents (e.g., NSAIDs, corticosteroids, cytokine inhibitors) directly to inflamed neural tissues (Wang 2018). Studies have shown that LNPs loaded with dexamethasone can effectively reduce neuroinflammatory markers and mitigate withdrawal severity in preclinical addiction models (Ning 2022). Gold nanoparticles (AuNPs) conjugated with anti‐inflammatory peptides and curcumin‐loaded polymeric nanoparticles have also demonstrated neuroprotective effects by suppressing glial cell activation, which contributes to withdrawal‐associated anxiety and depression (Chiang et al. 2024). Moreover, sustained‐release nanoparticle formulations allow for the gradual and prolonged release of anti‐inflammatory drugs, ensuring consistent therapeutic effects while reducing the need for repeated dosing (Placha and Jampilek 2021). This is particularly beneficial for opioid and stimulant withdrawal management, where long‐term neuroinflammation contributes to persistent cravings. These advancements underscore the potential of nanomedicine to revolutionize addiction treatment by providing precise, effective, and patient‐centered solutions.

## 4 SUD Pharmacogenomics and Nano‐Inspired Personalized Medicine

Pharmacogenomics has emerged as a transformative tool in understanding and addressing the genetic variability that underlies treatment responses in SUD. Advances in genetic research have revealed that variations in key genes, including OPRM1, CYP2D6, CYP3A4, ADH1B, DRD2, COMT, and FAAH, play pivotal roles in determining how individuals metabolize, respond to, and tolerate pharmacological interventions (Ettienne et al. 2017; Roden et al. 2011). These genetic variations influence drug efficacy, the likelihood of adverse effects, and long‐term treatment adherence. By incorporating these insights into clinical practice, pharmacogenomics provides a robust framework for tailoring treatments to individual genetic profiles, thereby enhancing therapeutic outcomes, minimizing side effects, and improving patient compliance through personalized medicine approaches.

Similarly, genes like CYP2D6 and CYP3A4, which encode enzymes in the cytochrome P450 family, profoundly impact the metabolism of opioid agonists such as methadone and buprenorphine (Fonseca Casals 2010). These enzymes dictate plasma drug concentrations, influencing therapeutic outcomes and safety profiles. Patients classified as poor metabolizers, for example, may require adjusted dosing regimens to avoid toxic levels, whereas ultra‐rapid metabolizers often need higher doses to achieve the desired therapeutic effect (Woillard et al. 2017). The role of pharmacogenomics extends beyond opioid and alcohol use disorders to stimulant and cannabis addictions. Genes such as DRD2 and COMT, involved in dopamine signaling and metabolism, influence responses to stimulant‐targeting medications, including methylphenidate and modafinil (Śmiarowska et al. 2022). These variations determine the efficacy of these medications in modulating dopaminergic pathways critical for reward processing and craving reduction. Similarly, polymorphisms in FAAH, a gene implicated in endocannabinoid metabolism, play a central role in shaping the response to FAAH inhibitors used to manage cannabis withdrawal symptoms and cravings (Boileau et al. 2016). Recent advancements in nanomedicine have enabled the integration of pharmacogenomics with nano‐enabled drug delivery, providing a more precise and targeted approach to SUD treatment (Malsch 2005). Nanoparticles functionalized with genetic targeting ligands can optimize therapeutic delivery to addiction‐related pathways, reducing off‐target effects and enhancing treatment adherence.

The application of pharmacogenomics in SUD treatment also highlights its capacity to identify new therapeutic targets and guide combination regimens. For instance, genetic screening can optimize Poly‐pharmacological approaches, such as combining naltrexone with dopamine agonists in stimulant addiction or pairing buprenorphine with adjunctive agents for opioid dependence (Mannelli et al. 2012). These strategies, informed by genetic insights, address the multifaceted nature of addiction, targeting both the biological and psychosocial dimensions of the disorder. Table 4 provides a consolidated overview of these pharmacogenomic pathways, illustrating their associated drugs, SUD applications, and clinical implications. By summarizing how specific genetic markers influence drug metabolism and treatment responses, the table serves as a critical reference for clinicians seeking to integrate pharmacogenomics into personalized SUD care. It underscores the potential of genetic profiling to revolutionize addiction medicine, enabling more precise, effective, and patient‐centered therapeutic strategies.

**TABLE 4:** Pharmacogenomic pathways and their implications for personalized nano‐SUD therapies.

Gene: drug targets	SUD application	Pharmacogenomic insights	Treatment implications
**OPRM1:** Naltrexone	AUD, OUD	Certain alleles are associated with improved alcohol craving reduction and adherence.	Enables patient stratification for naltrexone therapy to enhance efficacy and minimize relapse.
**CYP2D6/CYP3A4** Buprenorphine and Methadone	OUD	Variants affect drug metabolism rates, leading to variations in plasma levels and therapeutic outcomes.	Guides dosage adjustments and drug selection to optimize treatment response and reduce toxicity risks.
**FAAH:** FAAH Inhibitors	CUD	Polymorphisms influence endocannabinoid metabolism, impacting responses to FAAH‐targeted therapies.	It tailors FAAH inhibitor therapy to genetic profiles for improved efficacy in managing cannabis withdrawal and cravings.
**COMT:** Dopamine Modulators	Stimulant Use Disorders	Polymorphisms alter dopamine metabolism, influencing the efficacy of stimulant‐targeting medications like methylphenidate.	Supports personalized therapy for stimulant addiction by matching drugs to individual dopamine pathway profiles.
**DRD2:** Dopamine Agonists, Vaccines	Stimulant and Opioid Use Disorders	Variations in dopamine receptor function impact craving reduction and reward response.	Enhances outcomes by combining dopamine‐targeted therapies with pharmacogenomic data for improved relapse prevention.

### 4.1 Combination Therapies Guided by Nanomedicine and Pharmacogenomics

Nanotechnology has transformed the implementation of pharmacogenomics in SUD treatment by enabling precision drug delivery to genetic and molecular targets implicated in addiction. Engineered nanoparticles provide a platform for enhancing the efficacy of pharmacogenomic‐guided therapies, addressing the challenges of drug delivery across the BBB and ensuring site‐specific action (Suri et al. 2007). On the other hand, pharmacogenomics has not only optimized single‐drug therapies but has also facilitated the development of combination regimens for SUDs. Recent advances in nanomedicine have enabled the incorporation of biomarkers, such as dopamine transporter density, opioid receptor expression, and inflammatory markers, to personalize treatment regimens for improved clinical outcomes (Mukherjee et al. 2020).

Gene therapy holds immense promise in addressing the genetic and epigenetic factors that contribute to addiction. Nanoparticles are now being utilized to deliver genetic materials, such as small interfering RNA (siRNA) and CRISPR‐Cas9 systems, directly to addiction‐related pathways (Paunovska et al. 2022; Zhang et al. 2020). For instance, gold nanoparticles have been employed to carry siRNA targeting dopamine transporters, effectively reducing drug‐seeking behavior in preclinical models (Jain et al. 2023). Additionally, exosome‐based nanocarriers have shown potential in delivering neurotrophic factors such as BDNF and GDNF to promote neuroprotection and neurodegeneration in addiction‐affected brain regions (Shetgaonkar et al. 2022). These advancements not only provide a novel approach to modulating addiction‐related genes but also open the door to personalized treatments tailored to an individual's genetic profile.

By identifying genetic markers that influence drug interactions and metabolic pathways, clinicians can design treatment protocols that target multiple mechanisms underlying addiction (Haile et al. 2009). For example, in opioid use disorder, patients with certain CYP2D6 polymorphisms may benefit from a combination of methadone and buprenorphine, where individualized dosing minimizes toxicity while maintaining efficacy (McCance‐Katz et al. 2010). In stimulant addiction, combination therapies targeting both dopamine and glutamate pathways have shown promise in addressing the complex neurochemical imbalances that drive addiction. For instance, patients with DRD2 polymorphisms benefit from regimens that pair dopamine agonists with glutamate modulators, reducing cravings and improving functional recovery (Markett et al. 2010). In alcohol addiction, genetic screening has guided the use of dual‐target therapies such as naltrexone combined with baclofen, which simultaneously modulate GABAergic and dopaminergic pathways (Lohoff 2020). Nanomedicine is further enhancing these approaches by enabling controlled and sustained drug release, improving adherence and reducing relapse rates.

Beyond traditional pharmacotherapy, nanotechnology has expanded the horizons of SUD treatment into cutting‐edge areas such as gene therapy and combination therapies, which further enhance treatment efficacy by addressing the multifaceted nature of SUDs. Nanomedicine‐based biosensors are being developed to monitor drug metabolism and treatment responses in real time, allowing clinicians to make data‐driven adjustments to therapy (Parvin et al. 2024). Nanoparticles can co‐encapsulate multiple therapeutic agents, allowing for the simultaneous targeting of diverse addiction pathways. One study demonstrated the effectiveness of nanoparticles co‐delivering opioid receptor modulators and dopamine agonists, which resulted in a 45% improvement in treatment outcomes for patients with co‐occurring opioid and stimulant use disorders (Volkow et al. 2019). Similarly, dendrimer‐based nanocarriers have shown potential in facilitating targeted delivery of neuroprotective agents, such as antioxidants and anti‐inflammatory molecules, to mitigate oxidative stress and neuroinflammation associated with chronic substance use (Paramanick et al. 2022).

Moreover, LNPs have been employed to encapsulate naltrexone, allowing for targeted delivery to addiction‐relevant brain regions, such as the nucleus accumbens, in individuals with OPRM1 variants (Spencer et al. 2023). This targeted approach enhances drug efficacy while minimizing systemic exposure, reducing the risk of side effects. Similarly, polymeric nanoparticles co‐delivering agents targeting genes such as FAAH and DRD2 offer a multifaceted approach to managing cannabis and stimulant addiction (Xia et al. 2021). These technologies enable simultaneous modulation of multiple pathways, addressing the complex genetic and neurochemical interactions underlying addiction.

### 4.2 Integrating Nanomedicines With Other Emerging SUD Therapies

Nanomedicine has demonstrated significant potential to transcend traditional pharmacological applications by seamlessly integrating with behavioral and device‐based therapies, thereby offering a more holistic framework for the treatment of SUD. The development of hybrid delivery systems exemplifies this integration, as these systems combine pharmacological interventions with neurostimulation devices to simultaneously address the neurochemical and behavioral aspects of addiction (Habelt et al. 2020). Recent studies have shown that such hybrid systems can significantly reduce relapse rates, achieving this by providing synchronized interventions that target both neural and behavioral pathways (Uhl et al. 2019). Advancements in neurostimulation techniques, such as transcranial magnetic stimulation (TMS) and deep brain stimulation (DBS), have shown promise in modulating addiction‐related neural circuits (Li et al. 2019). Recent studies have demonstrated that combining DBS with nanomedicine‐assisted drug delivery can enhance the precision of neuromodulation, leading to improved long‐term SUD treatment outcomes (Voges et al. 2013). MENPs have emerged as a promising technology to enhance DBS precision by enabling wireless control of neuronal activity.

Nanotechnology has also played a pivotal role in advancing nicotine vaccines. By employing LNPs to encapsulate nicotine antigens, researchers have enhanced vaccine efficacy by improving antigen stability and delivery mechanisms. Preclinical trials have reported up to a 50% reduction in brain nicotine levels, underlining the potential of this approach as a transformative treatment for tobacco use disorder (Benowitz 2009). Compared to traditional protein‐based or viral vector vaccines, LNP‐based vaccines provide several advantages, including improved antigen stability, enhanced immune response activation, and controlled release (Lin et al. 2024). Traditional nicotine and cocaine vaccines rely on protein conjugates that require multiple booster doses due to rapid antigen degradation. In contrast, LNP formulations protect antigens from enzymatic breakdown and facilitate prolonged immune activation, reducing the need for frequent dosing (Wang et al. 2024).

**FIGURE 6:**
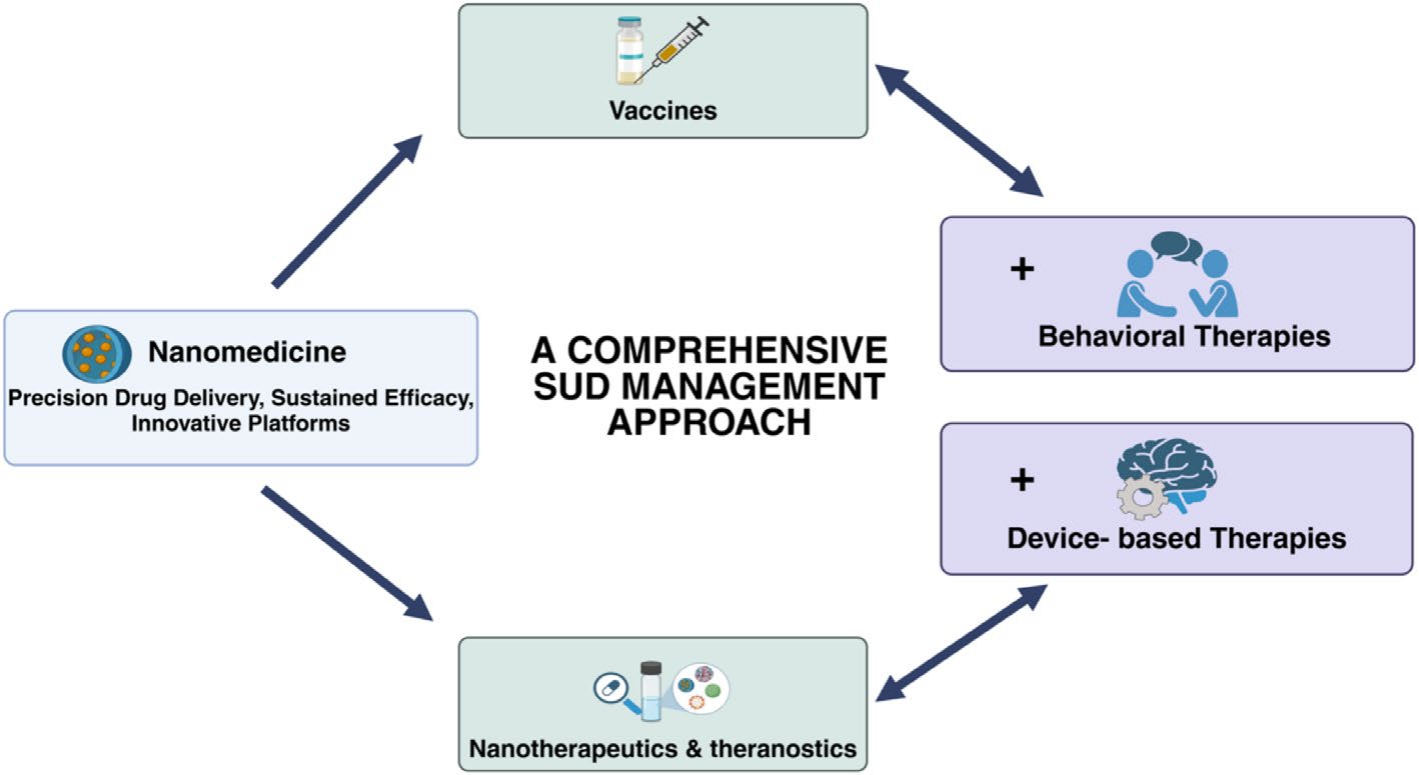
Nanomedicine‐driven hybrid solutions for a comprehensive SUD management.

Further, the integration of nanomedicine with optogenetic techniques is being explored to achieve selective modulation of addiction‐relevant neural pathways. Nanoparticle‐based optogenetic stimulation offers a precise, minimally invasive approach to controlling dopaminergic signaling, which is critical for addiction relapse and craving reduction (Balbinot et al. 2024). The integration of these nanoscale innovations with emerging therapies represents a crucial step in addressing the limitations of current treatment paradigms for SUDs. This multidisciplinary strategy not only enhances the precision and efficacy of therapeutic interventions but also lays the groundwork for more individualized and comprehensive care models tailored to the complexities of addiction (Malik et al. 2023a). Figure 6 visually encapsulates this synergy by highlighting the role of nanomedicine in enabling hybrid therapeutic solutions, thereby emphasizing its transformative impact on SUD management.

## 5 Nano‐Inspired Pharmacotherapy Addressing Barriers to Effective SUD Treatment

The integration of nanotechnology into SUD treatment represents a groundbreaking approach to overcoming barriers that have long impeded recovery. Socioeconomic challenges, ethical complexities, and the evolving landscape of novel interventions are significant hurdles (Kasina et al. 2022). With its precise targeting and innovative delivery platforms, nanomedicine addresses key barriers in addiction treatment, improving both access and therapeutic effectiveness.

### 5.1 Technological Barriers

Research on nanotechnology‐based targeting for SUD faces several significant challenges. First, the BBB is a highly selective barrier that prevents most substances from entering the brain. Designing nanoparticles that can effectively cross the BBB without causing damage is a major hurdle (Mohamed 2024). Nanoparticles are now being engineered with active transport mechanisms, including ligand‐functionalized nanoparticles (e.g., transferrin, lactoferrin, and glucose transporters) to enhance BBB penetration (Lahkar and Das 2019). Additionally, magnetoelectric nanoparticles and exosome‐mimicking nanocarriers are emerging as novel strategies for targeted CNS drug delivery. Second, some materials used in nanoparticles can cause biocompatibility issues and adverse reactions or long‐term toxicity, which limits their clinical application (Kyriakides et al. 2021). Advancements in biodegradable and biocompatible nanomaterials, such as polymeric nanoparticles and lipid‐based nanocarriers, are addressing these toxicity concerns (Farasati Far et al. 2023). Moreover, AI‐driven predictive models are optimizing nanoparticle designs to minimize immunogenicity and enhance therapeutic outcomes. Third, achieving precise targeting to specific brain regions or cells involved in addiction is challenging. Non‐specific distribution of nanoparticles can lead to off‐target effects and reduced therapeutic efficacy (Kakinen et al. 2024). A major challenge in the clinical translation of nanomedicine is the scalability of nanoparticle production. Traditional batch synthesis methods suffer from variability and reproducibility issues, affecting nanoparticle consistency. To address this, emerging microfluidics‐based synthesis allows for high‐throughput and precise control over nanoparticle formulation, ensuring uniformity in size, shape, and drug loading (Ahn et al. 2018). This technique enhances reproducibility and reduces production costs, facilitating large‐scale manufacturing for clinical applications. Additionally, AI‐driven quality control systems are being integrated into nanoparticle production pipelines to optimize batch consistency, predict stability, and minimize production failures. Automated high‐throughput screening further accelerates the development of nanomedicine formulations, allowing for faster preclinical‐to‐clinical transition. Cost‐effectiveness remains a critical barrier to clinical translation. The initial development costs for nanomedicine‐based therapies are high due to the need for specialized materials, production facilities, and regulatory approvals (Foulkes et al. 2020). However, the long‐term benefits, including reduced treatment frequency and improved therapeutic efficacy, may offset these costs. Public‐private partnerships and regulatory incentives can further promote the commercialization of scalable nanomedicine solutions. Fifth, since producing nanoparticles consistently and at a scale suitable for clinical use is difficult, any variability in nanoparticle production can affect their performance and safety. By integrating microfluidic production, AI‐driven quality assurance, and scalable synthesis techniques, nanomedicine can move toward cost‐effective and clinically viable solutions (Agrahari et al. 2024). Finally, the regulatory landscape for nanomedicine is complex. There are also ethical considerations regarding the long‐term effects and potential misuse of nanotechnology in treating SUDs. Whereas these challenges are not specific to SUDs, addressing these challenges is essential for advancing the field and developing effective nanotechnology‐based treatments for SUDs.

### 5.2 Socioeconomic Barriers

Socioeconomic factors remain among the most critical determinants of treatment outcomes in SUD management, as they directly impact a patient's ability to adhere to therapeutic regimens and maintain long‐term recovery. Financial instability, housing insecurity, unemployment, and lack of access to healthcare create a cycle that perpetuates addiction and relapse. Nano‐inspired pharmacotherapies, by leveraging advances in drug delivery and affordability, offer opportunities to mitigate these challenges.

Innovative nanotechnology‐based therapies, including extended‐release nanoparticles, address adherence challenges by reducing treatment frequency and increasing accessibility for underserved populations. For example, LNPs delivering opioid receptor antagonists like naltrexone have demonstrated efficacy in providing sustained‐release profiles for up to several weeks (Santos et al. 2020). This reduces the need for frequent clinical visits, making treatment more feasible for individuals with socioeconomic constraints.

Additionally, the integration of pharmacotherapies with community support programs is essential. Nano‐inspired therapies can be embedded into holistic care models that combine medical interventions with social services, offering patients stable housing, employment assistance, and access to financial support. Programs like contingency management, which incentivize adherence through monetary rewards or vouchers, can be augmented by nanotechnology's precision delivery systems, ensuring effective therapeutic outcomes with minimal logistical complexity.

Policymakers and healthcare providers must collaborate to develop frameworks that subsidize the cost of advanced therapies for underserved populations. Public funding initiatives or partnerships with private organizations could reduce out‐of‐pocket expenses for patients, ensuring that socioeconomically disadvantaged groups can benefit from nano‐inspired pharmacotherapies. By addressing these barriers, nanotechnology not only improves individual outcomes but also reduces the broader societal costs of addiction, including those related to healthcare and criminal justice systems.

### 5.3 Ethical Considerations

As the field of pharmacotherapy evolves with the advent of innovative treatments such as nanotechnology, immunotherapy, and neurostimulation, ethical and regulatory challenges become increasingly complex. Nano‐inspired therapies, while offering immense potential, require careful consideration to balance innovation with safety and equity.

The use of nano‐based therapies in SUD treatment raises questions about patient autonomy, informed consent, and the equitable distribution of experimental treatments. Many patients with SUDs belong to vulnerable populations, including those with limited education, mental health conditions, or unstable living environments. Ensuring that these individuals fully understand the benefits and risks of novel treatments is paramount. For instance, patients must be adequately informed about the long‐term effects of nanotechnology‐based interventions, such as polymeric nanoparticles delivering neurostimulation agents, which may involve risks that are not immediately evident during early trials.

Additionally, ethical frameworks must prevent exploitation of vulnerable groups in clinical trials for nano‐inspired therapies. Clear guidelines on participant selection, compensation, and post‐trial care are necessary to protect patient rights and ensure that benefits are equitably shared among all populations, rather than being limited to those with financial or geographic access to cutting‐edge treatments.

### 5.4 Regulatory Adaptations

The rapid pace of innovation in nanotechnology necessitates updates to existing regulatory frameworks to ensure that new therapies are both safe and accessible. Traditional drug approval processes, which often involve prolonged clinical trials, may be insufficient for evaluating the unique mechanisms of nano‐based therapies. For example, nanoparticles' ability to cross the BBB and deliver drugs directly to neural circuits implicated in addiction requires new testing protocols that assess long‐term neurotoxicity, biodegradability, and off‐target effects (Kulkarni et al. 2024).

Regulatory agencies must adopt adaptive review processes that incorporate real‐world evidence and post‐market surveillance to ensure that nano‐inspired treatments meet rigorous safety standards while expediting access for patients in need. Collaboration between regulatory bodies, academic researchers, and industry stakeholders is critical to developing comprehensive guidelines for evaluating these therapies. Proactive regulatory policies, such as fast‐track designations or conditional approvals, can foster innovation while maintaining patient safety.

## 6 Future Directions and Challenges in SUD Treatment

The future of SUD treatment lies in overcoming existing barriers to care while harnessing the potential of nano‐inspired pharmacotherapies to create scalable, cost‐effective, and patient‐centered solutions. These innovations, combined with interdisciplinary collaboration and focused research on underexplored areas, hold the promise of revolutionizing addiction medicine by making advanced treatments more accessible and equitable.

### 6.1 Fostering Cross‐Disciplinary Collaboration

The successful development and implementation of advanced SUD treatments require a collaborative approach that bridges multiple disciplines. Pharmacologists, material scientists, and biomedical engineers must work closely with behavioral scientists and clinicians to design therapies that address both the biological and psychological aspects of addiction. For instance, the integration of nano‐based pharmacological agents with behavioral interventions and digital health tools can create comprehensive treatment solutions tailored to individual needs (Haleem et al. 2023).

Partnerships between academia, industry, and regulatory agencies are essential for translating research into practical applications. For example, pharmaceutical companies can leverage nanotechnology breakthroughs from academic institutions to develop multimodal therapies that combine nano‐inspired pharmacotherapies with cognitive‐behavioral therapy (CBT) or neurostimulation devices (Malik et al. 2023a). Successful interdisciplinary collaborations have played a crucial role in advancing innovative pharmacotherapies for addiction treatment. The National Institutes of Health (NIH) Helping to End Addiction Long‐term (HEAL) Initiative has fostered partnerships between academic researchers, pharmaceutical companies, and public health agencies to accelerate the development of novel opioid addiction therapies (NIH HEAL: https://heal.nih.gov/). Similarly, the European Commission's Nanomedicine Initiative has demonstrated the effectiveness of cross‐disciplinary research in facilitating nanoparticle‐based drug delivery systems for neurological disorders, including addiction‐related conditions. These models provide a framework for fostering future collaborative efforts in the translation of nanomedicine for SUD treatment.

Cross‐sector collaborations can also address the challenge of integrating nanomedicine into diverse healthcare settings. By pooling resources and expertise, stakeholders can create standardized protocols for the administration of nano‐based therapies, ensuring consistency in treatment delivery across urban and rural regions alike.

#### 6.1.1 Enhancing Accessibility and Equity Through SUD Nanomedicines

Nanomedicine presents a unique opportunity to make advanced therapies for SUDs more affordable and widely available. By optimizing drug formulations and delivery systems, nanotechnology can reduce manufacturing costs and streamline the production of medications. For example, nanoparticle‐based sustained‐release formulations can decrease the frequency of dosing, reducing the logistical and economic burden on healthcare systems while ensuring consistent therapeutic effects (Ezike et al. 2023).

Advancements in nanotechnology also allow for scalable production processes that leverage cost‐efficient materials, such as biodegradable polymers and lipid‐based carriers. These materials are not only biocompatible but also readily available, enabling large‐scale manufacturing at a fraction of the cost of traditional pharmaceuticals (Malik et al. 2023b). Additionally, the high precision of nanomedicine minimizes wastage of active pharmaceutical ingredients, further contributing to cost reductions.

Collaborative efforts between research institutions, pharmaceutical companies, and government agencies can accelerate the development of generic nanomedicine formulations. Generic options significantly lower the cost of treatment by increasing market competition while maintaining the efficacy of branded therapies (Halwani 2022). By integrating nanomedicine into public health frameworks and leveraging economies of scale, these therapies can become an integral part of standard SUD care, ensuring that they reach a broader population.

Moreover, advances in nanotechnology enable the development of long‐acting injectable formulations and implantable devices that deliver medications over extended periods. These innovations eliminate the need for frequent visits to healthcare facilities, reducing indirect costs such as transportation and time off work for patients. Such solutions not only make treatments more accessible but also enhance adherence and long‐term efficacy.

Policymakers and healthcare stakeholders can further support this transition by funding nanotechnology research focused on creating low‐cost manufacturing pipelines. Public‐private partnerships can play a pivotal role in establishing production facilities dedicated to affordable nanomedicine, ensuring that even the most advanced therapies are accessible to diverse populations.

### 6.2 Interdisciplinary Collaborations and Policy Reforms

The integration of nanomedicine into SUD treatment necessitates a coordinated effort among scientists, policymakers, healthcare providers, and industry leaders. Effective interdisciplinary collaborations have played a crucial role in advancing innovative pharmacotherapies for addiction treatment. Notable initiatives such as the National Institutes of Health (NIH) Helping to End Addiction Long‐term (HEAL) Initiative have fostered partnerships between academic researchers, pharmaceutical companies, and public health agencies to accelerate the development of novel opioid addiction therapies (NIH, HEAL initiative). Similarly, the European Commission's Nanomedicine Initiative has demonstrated the effectiveness of cross‐disciplinary research in facilitating nanoparticle‐based drug delivery systems for neurological disorders, including addiction‐related conditions (https://projects.research‐and‐innovation.ec.europa.eu/en/projects/success‐stories/all/nanomedicine).

To ensure that nanomedicine‐based interventions transition from laboratory research to widespread clinical application, several key strategies must be implemented. One critical approach is the expansion of public‐private partnerships to support sustained investment in nanotechnology research for SUD treatment (Hunter et al. 2016). By facilitating collaborations between government agencies, pharmaceutical industries, and biotechnology firms, these partnerships can drive the development of targeted therapies that address the complex neurobiological mechanisms underlying addiction. Additionally, the establishment of standardized protocols for nanoparticle‐based therapies is essential to ensure consistency in clinical applications, particularly for formulations designed to cross the blood–brain barrier (BBB) and precisely target addiction‐relevant brain regions.

Regulatory frameworks must also evolve to accommodate the emerging field of nano‐inspired pharmacotherapies. The development of fast‐track approval pathways for promising nanomedicine‐based treatments would enable more efficient translation of research findings into clinical practice (Đorđević et al. 2022). Countries such as Canada have already implemented policy reforms that facilitate regulatory approvals for nanoparticle‐based opioid addiction treatments, leading to significant reductions in patient relapse rates. Similarly, the U.S. Food and Drug Administration (FDA) has introduced regulatory pathways tailored for nanomedicine‐based therapeutics, ensuring that these novel interventions undergo rigorous evaluation while expediting their clinical adoption (Paradise 2019).

Beyond regulatory advancements, there is a pressing need for policies that incentivize healthcare providers to integrate nano‐based pharmacotherapies into existing SUD treatment frameworks (Janero 2014). Financial incentives, such as tax benefits or subsidies for healthcare institutions that adopt nanomedicine‐based treatment protocols, could accelerate the acceptance of these innovations. Moreover, educational programs targeting clinicians, researchers, and policymakers can increase awareness of the potential benefits of nanotechnology in addiction medicine, thereby fostering broader acceptance and implementation. Additionally, regulatory agencies should establish clear safety guidelines for nano‐inspired pharmacotherapies, including long‐term toxicity assessments and pharmacokinetic profiling specific to nanoparticle‐based interventions (Ma et al. 2024). Implementing adaptive regulatory approaches that incorporate real‐world evidence and post‐market surveillance will ensure both safety and efficiency in clinical applications.

Successful interdisciplinary collaborations and policy reforms have the potential to revolutionize SUD treatment by ensuring that nano‐inspired pharmacotherapies are not only scientifically viable but also accessible and effectively integrated into real‐world healthcare settings (Kools et al. 2022). By adopting a proactive approach that includes sustained research funding, regulatory adaptability, and comprehensive education initiatives, the widespread adoption of nanomedicine for addiction treatment can become a reality.

#### 6.2.1 Expanding Research in Understudied Areas

While substantial progress has been made in treating opioid and alcohol use disorders, other SUDs, such as stimulant and cannabis use disorders, remain underexplored. Nanotechnology offers an opportunity to advance research in these areas by enabling the precise targeting of addiction‐relevant pathways (Bozkurt 2022). For example, nanoparticles engineered to deliver dopamine or glutamate modulators can provide novel solutions for stimulant addiction, while lipid‐based nanoparticles targeting the endocannabinoid system hold promise for cannabis use disorder (Maccarrone et al. 2023).

Future research should also focus on developing innovative therapies that address co‐occurring conditions, such as SUDs coupled with mental health disorders. Through its innovative platforms, nanomedicine enables dual‐agent delivery, targeting addiction alongside mental health conditions such as anxiety or depression for a more holistic treatment approach (Zorkina et al. 2020).

Another critical area of investigation is the long‐term efficacy and safety of nano‐inspired pharmacotherapies. Conducting large‐scale, longitudinal studies will ensure that these treatments are both effective and sustainable across diverse populations. Research must also prioritize the development of robust diagnostic tools, such as biosensors and nanotechnology‐enhanced imaging, to improve the early detection and monitoring of addiction (Marsch 2012).

#### 6.2.2 Scalability and Manufacturing Challenges

While nanotechnology offers promising advancements in SUD treatment, scaling up production and ensuring consistency across manufacturing processes present significant hurdles. One of the primary concerns is the high cost of nanoparticle production, which remains a major barrier to widespread clinical adoption. To address this challenge, researchers and manufacturers are exploring cost‐effective alternatives such as biodegradable polymers and lipid‐based carriers (Cardoso et al. 2016). These materials not only reduce production expenses but also ensure biocompatibility and efficacy, making them viable options for large‐scale manufacturing.

Another critical issue is batch‐to‐batch consistency in nanoparticle production. Small variations in particle size, surface properties, or drug loading can significantly impact therapeutic outcomes, posing challenges for regulatory approval and clinical application. Microfluidics‐based synthesis has emerged as a promising solution to this problem. By offering precise control over particle characteristics, microfluidics improves reproducibility and scalability, ensuring consistent quality across different production batches (Alavi et al. 2024).

Moving from preclinical studies to clinical trials presents another set of challenges. Many nano‐inspired pharmacotherapies face delays due to complex regulatory approval requirements and the need for large‐scale production facilities that adhere to Good Manufacturing Practice (GMP) standards (Souto et al. 2020). The transition from laboratory research to practical application is often hindered by the lack of infrastructure to support mass production and the stringent regulations governing nanomedicine therapies.

To overcome these obstacles, researchers are turning to emerging solutions such as microfluidics, 3D printing, and automated synthesis. Microfluidic platforms enable continuous nanoparticle production with high precision, significantly reducing batch variability. Similarly, 3D printing is revolutionizing drug delivery by facilitating the design of personalized treatment systems, allowing for patient‐specific therapies tailored to individual needs (Alzoubi et al. 2023). Automated synthesis further enhances manufacturing by minimizing human error, improving efficiency, and streamlining the large‐scale production of nano‐based therapeutics.

Investing in these advanced manufacturing technologies is essential to making nano‐based therapies both affordable and widely accessible. By integrating scalable solutions into existing production frameworks, the pharmaceutical industry can ensure that nanomedicine plays a transformative role in the future of SUD treatment.

### 6.3 Bridging Innovation and Real‐World Application

To ensure that the benefits of nanomedicine reach all individuals affected by SUDs, efforts must focus on bridging the gap between innovation and practical implementation. This involves designing scalable, patient‐centered solutions that can be seamlessly integrated into existing healthcare systems. For example, the development of user‐friendly delivery systems, such as wearable devices or implantable drug reservoirs, can enhance patient compliance and reduce reliance on complex treatment protocols (Ghanim et al. 2023).

Scalability is key to making nano‐based therapies widely available. By leveraging manufacturing technologies such as microfluidics and 3D printing, the production of nanoparticles can be automated and standardized, significantly reducing costs while ensuring consistent quality (Rahman et al. 2024). These advancements pave the way for the mass production of nanomedicine, making it feasible to distribute these therapies globally.

Public health initiatives should prioritize the inclusion of nanomedicine in treatment guidelines for SUDs. Policymakers can incentivize the adoption of these therapies by offering subsidies or tax benefits to healthcare providers and manufacturers. At the same time, education campaigns targeting clinicians and patients can raise awareness about the advantages of nano‐inspired pharmacotherapies, fostering acceptance and uptake.

## 7 Concluding Remarks

The treatment of SUD has seen remarkable advancements, driven by breakthroughs in pharmacotherapy, behavioral interventions, and cutting‐edge technologies such as nanomedicine and neurostimulation. These innovations underscore a growing understanding of addiction as a multifaceted condition encompassing neurobiological, psychological, and social dimensions. While these advancements offer significant promise, the path to widespread and effective clinical application remains complex. Overcoming barriers such as accessibility, systemic inequities, and persistent stigma is essential to realizing the full potential of these innovations.

Pharmacotherapy has emerged as a cornerstone of SUD treatment, offering profound potential to mitigate cravings and prevent relapse. However, limitations in equitable access continue to constrain its broader impact, particularly in rural and underserved communities. The integration of novel technologies like machine learning, including wearable biosensors, digital therapeutics, and gene‐targeted therapies, offers opportunities to enhance precision, adherence, and long‐term outcomes (Sabry et al. 2022; Tripathy et al. 2024). Yet, for these advancements to achieve their intended impact, they must be delivered within a framework that ensures affordability, scalability, and equitable distribution across diverse populations.

A holistic, patient‐centered approach remains vital as the treatment paradigm continues to evolve. Effective SUD management transcends neurobiological interventions by addressing the broader psychosocial determinants of health. Embedding pharmacotherapy within comprehensive care models that integrate behavioral support, community‐based interventions, and digital health tools can pave the way for more sustainable and personalized recovery pathways (Bowen et al. 2020). Such models recognize that addressing the external factors influencing addiction, such as social support and economic stability, is as important as targeting its internal mechanisms.

Collaboration across disciplines will be a cornerstone of future progress in SUD treatment. Partnerships between researchers, clinicians, policymakers, and industry leaders are essential for fostering the development and implementation of innovative therapies (Baldwin et al. 2020). These collaborations can also drive inclusive research efforts that reflect the genetic, cultural, and socioeconomic diversity of individuals affected by SUD. Advancing the field will further require robust policy frameworks that bridge gaps in care, coupled with public health initiatives to promote early intervention and reduce the stigma surrounding addiction. Education campaigns aimed at increasing awareness and understanding of SUD as a treatable condition will be instrumental in creating a supportive environment for those seeking care.

Leveraging insights from pharmacogenomics and personalized medicine offers the potential to refine treatments for individual needs. By tailoring therapies to patients' unique genetic and biological profiles, healthcare providers can achieve greater precision and effectiveness. The vision of transforming addiction care into a more individualized, accessible, and sustainable model is within reach. Achieving this goal will depend on sustained investment in research, equitable resource distribution, and the integration of multidisciplinary approaches.

The promise of a transformative era in SUD treatment highlights the importance of prioritizing innovation, equity, and collaboration. By uniting efforts across all sectors of society, effective and individualized care can become a standard of practice rather than an exception (Chuang et al. 2024). Progress in this area will not only alleviate the profound burden of addiction on individuals and their families but also enhance the overall health, productivity, and resilience of communities. With continued dedication to advancing research, improving accessibility, and fostering interdisciplinary approaches, recovery can become a realistic and achievable goal for everyone affected by SUDs. This transformative vision underscores the need for sustained commitment and inclusive practices as we strive toward a more equitable and effective healthcare landscape.

## Author Contributions

**Akshata Y. Patne:** data curation (supporting), methodology (supporting), project administration (supporting), supervision (equal), validation (supporting), visualization (supporting), writing – original draft (lead). **Subhra Mohapatra:** conceptualization (lead), data curation (lead), formal analysis (lead), funding acquisition (lead), investigation (lead), methodology (lead), project administration (lead), supervision (lead), validation (lead), visualization (lead), writing – review and editing (lead). **Shyam S. Mohapatra:** conceptualization (equal), data curation (equal), funding acquisition (equal), investigation (lead), project administration (equal), resources (lead), supervision (lead), validation (lead), writing – review and editing (lead).

## Conflicts of Interest

The authors declare no conflicts of interest.

## Related WIREs Articles

A systematic review and meta‐analysis of neuromodulation therapies for substance use disorders.

## Data Availability

All other data generated or analyzed during this study are included in this published article (and its Supporting Information).
